# Quality of life in cancer patients undergoing chemotherapy: physical, psychological, and social determinants

**DOI:** 10.1192/j.eurpsy.2026.10626

**Published:** 2026-07-31

**Authors:** A. M. Cybulska, I. Bułatowicz, K. Rachubińska, A. Królikowska, A. Derezińska, D. Schneider-Matyka

**Affiliations:** 1 Department of Nursing; 2 Science Club at Department of Nursing, Pomeranian Medical University in Szczecin, Szczecin, Poland

## Abstract

**Introduction:**

Quality of life in oncology is a multidimensional construct encompassing physical, psychological, and social domains, particularly in advanced stages of disease. Cancer patients often experience pain, fatigue, and prolonged exhaustion, limiting daily functioning. Coping with the trauma of diagnosis, side effects of treatment, and maintaining psychosocial stability are crucial challenges. Supportive medical, psychological, rehabilitative, and palliative care, along with family and professional support, play a key role in improving wellbeing during chemotherapy.

**Objectives:**

The primary objective was to assess the quality of life of cancer patients undergoing chemotherapy and to identify sociodemographic and clinical factors influencing its different domains.

**Methods:**

A cross-sectional study was conducted among 226 oncology patients undergoing chemotherapy in the West Pomeranian Voivodeship, Poland. Data were collected using a diagnostic survey method, which included an original questionnaire and standardized Polish versions of validated research tools: the European Organization for Research and Treatment of Cancer Quality of Life Questionnaire (EORTC QLQ-C30), the Functional Assessment of Cancer Therapy–General (FACT-G), and the Depression, Anxiety, and Stress Scale (DASS-21).

**Results:**

Most patients received intravenous chemotherapy (83.09%), usually for less than five years and up to 20 cycles. Quality of life scores varied significantly by age, education, marital status, and residence. The patients treated for less than one year reported better physical and functional wellbeing compared to those treated for 1–5 years or more than 10 years. Higher numbers of chemotherapy cycles correlated with better social functioning but also increased dyspnea. The respondents with colorectal and breast cancer achieved higher social and functional wellbeing compared to those with bladder, cervical, or renal cancers. Commonly reported symptoms negatively affecting quality of life included pain, fatigue, irritability, and helplessness. A significant negative correlation was observed between depression, anxiety, and stress and functional/social wellbeing.

**Conclusions:**

The chemotherapy patients reported reduced quality of life, mainly due to insomnia, fatigue, and appetite loss. Shorter treatment duration and oral regimens were linked to better outcomes, while comorbidities and long-term therapy worsened them. Depression, anxiety, and stress strongly correlated with lower physical, emotional, and social functioning, with colorectal and breast cancer patients showing relatively higher wellbeing.

**Disclosure of Interest:**

None Declared

